# STAT6-Dependent Collagen Synthesis in Human Fibroblasts Is Induced by Bovine Milk

**DOI:** 10.1371/journal.pone.0131783

**Published:** 2015-07-02

**Authors:** Stefan Kippenberger, Nadja Zöller, Johannes Kleemann, Jutta Müller, Roland Kaufmann, Matthias Hofmann, August Bernd, Markus Meissner, Eva Valesky

**Affiliations:** Department of Dermatology, Venereology and Allergy, Johann Wolfgang Goethe University, Frankfurt/Main, Germany; San Gallicano Dermatologic Institute, ITALY

## Abstract

Since the domestication of the *urus*, 10.000 years ago, mankind utilizes bovine milk for different purposes. Besides usage as a nutrient also the external application of milk on skin has a long tradition going back to at least the ancient Aegypt with Cleopatra VII as a great exponent. In order to test whether milk has impact on skin physiology, cultures of human skin fibroblasts were exposed to commercial bovine milk. Our data show significant induction of proliferation by milk (max. 2,3-fold, EC50: 2,5% milk) without toxic effects. Surprisingly, bovine milk was identified as strong inducer of collagen 1A1 synthesis at both, the protein (4-fold, EC50: 0,09% milk) and promoter level. Regarding the underlying molecular pathways, we show functional activation of STAT6 in a p44/42 and p38-dependent manner. More upstream, we identified IGF-1 and insulin as key factors responsible for milk-induced collagen synthesis. These findings show that bovine milk contains bioactive molecules that act on human skin cells. Therefore, it is tempting to test the herein introduced concept in treatment of atrophic skin conditions induced e.g. by UV light or corticosteroids.

## Introduction

Domestication of the *urus* in Eurasia took place in the Pre-Pottery Neolithic. Besides the cultivation of land, cattle husbandry was mainly exploited for the production of meat and milk. In addition to fresh milk, cultural techniques created a plethora of milk-derived products such as cheeses and yoghurts serving the nutrition of man. Although, milk is generally associated with a positive image there is increasing evidence that enteral consumption of milk may be a trigger factor for some diseases. What sure is, milk, and particularly colostrums, supports health in many neonates including humans. Particularly, the supply with milk-derived immunoglobulins is known to reduce infections in newborns [1]. Excluding the period of breastfeeding, milk, and milk-derived products, consumed by humans are mainly from bovine source. Now, there is debate about the relevance of bioactive factors present in bovine milk for human health [2]. In regard to human skin large clinical trials have shown a correlation between milk consumption and acne vulgaris [3, 4]. Corroboratively, recent studies found that high milk intake is associated with other health disorders. In this respect an elevated mortality and, most surprisingly, also a higher facture incidence, was found [5]. Moreover, diet with milk and dairy products is suspected to contribute to male reproductive disorders because of the levels of estrogen therein [6]. On the other side there is evidence that, at least factors contained in milk, have beneficial effects on human health. For example conjugated linoleic acid (CLA), a trans-fatty acid, offers anticarcinogenic, antiatherogenic and antidiabetogenic properties [7–9].

Besides enteral use, external application of milk and milk components look back on a history of several thousand years. Tradition has it that Cleopatra VII, Elizabeth I of England, Elisabeth (“Sisi”) of Bavaria and others have bathed in milk for beautifying benefits. And still today cosmetic products, containing milk-derived ingredients, are marketed for skin care. In addition to this, polar lipids from bovine milk have been shown to penetrate the intact skin inducing hair cycle progression in a mouse model [10]. Moreover, expressed maternal milk seems to alleviate nipple pain in breastfeeding women [11]. Interestingly, also clinical conditions such as papillomas benefit from a topical treatment with a α-lactalbumin preparation extracted from human milk as shown by a clinical trial [12].

The aforementioned examples inspired the present study to test whether bovine milk has impact on the physiology of dermal fibroblasts. Most interestingly, we found upregulation of proliferation and collagen synthesis by bovine milk. These results provide a scientific rational for treatment of atrophic skin with topical preparations containing milk or milk-derived factors.

## Materials and Methods

### Ethics Statement

This study was conducted according to the Declaration of Helsinki Principles and in agreement with the Local Ethic Commission of the faculty of Medicine of the Johann Wolfgang Goethe University (Frankfurt am Main, Germany). The Local Ethic Commission waived the need for consent.

### Reagents and antibodies

Fresh bovine milk (Weihenstephan, 3.5% fat) was aliquot and stored until use at -20°C. Insulin, IGF-1 and testosterone were purchased from Sigma-Aldrich (Taufkirchen, Germany), TGFβ1 was from R&D Systems (Wiesbaden, Germany). The following neutralizing antibodies were used: anti-IGF-1 (#5119–100, BioVision, Heidelberg, Germany), anti-TGFβ1,2,3 (MAB1835, R&D Systems) and anti-insulin receptor (MA-20, Novus Biologicals, Cambridge, UK). The MEK1 inhibitor PD98059 was from Cell Signaling (Frankfurt, Germany). PI3K inhibitor LY-294002, EGF receptor tyrosine kinase inhibitor AG1478 and p38 inhibitor SB203580 were purchased from Calbiochem-Novabiochem (Darmstadt, Germany). Leflunomide to inhibit signal transducers and activators of transcription 6 (STAT6) signalling [13] was from Sigma-Aldrich. For Western blotting, activation of signalling molecules was detected with primary phosphospecific antibodies against PKB/Akt (Ser473/Thr308), p44/42 (Thr202/Tyr204), p38 (Thr180/Tyr182) (all from Cell Signaling Technology) and pSTAT6 (Tyr641) (from Santa Cruz Biotechnology, Heidelberg, Germany). Equal loading was controlled with an antibody against total PKB/Akt (Cell Signaling Technology) and β-actin (Sigma-Aldrich).

### Cell culture

Normal human skin fibroblasts were isolated either from preputia (or from abdominal skin for control experiments, S1 Fig). If not otherwise indicated experiments were performed with preputial fibroblasts. Cells were propagated in RPMI 1640 medium (Biochrom, Berlin, Germany) with 10% FCS (PAA, Cölbe, Germany) and 1% penicillin-streptomycin solution (Biochrom KG, Berlin, Germany) at 37°C in a 5% CO_2_-atmosphere. The medium was renewed twice a week. All experiments were done in agreement with the local ethics commission.

### DNA synthesis

Cells were cultivated in microwell plates at a density of 2x10^4^ cells/0.33 cm^2^. Cells were exposed for 24 hours to bovine milk at the indicated concentrations. For the last 24 hours cells were pulsed with 5-bromo-2′-deoxyuridine (BrdU). Subsequently, the incorporation rate of BrdU was determined using a commercial enzyme-linked immunosorbent assay kit (Roche, Mannheim, Germany). Briefly, cells were fixed and immune complexes were formed using peroxidase-coupled BrdU-antibodies. A colorimetric reaction with tetramethylbenzidine as a substrate gives rise of a reaction product measured at 450 nm in a scanning multiwell spectrophotometer (ELISA reader, MR 5000, Dynatech, Guernsey, UK).

### Membrane integrity

Cell lysis was quantified using the cytotoxicity detection kit (Roche), which is based on the release of lactate dehydrogenase (LDH) from damaged cells. Briefly, cells were seeded out in microwell plates at a density of 2x10^4^ cells/0.33 cm^2^ and treated with bovine milk at the indicated concentrations for 24 hours. Consecutively, the cell-free supernatants were incubated with NAD+, which becomes reduced by LDH to NADH/H+. In a second step NADH/H+ reduces a yellow tetrazolium salt to a red-coloured formazan salt. The amount of red colour is proportional to the number of lysed cells. For quantitation, the absorbance of the reaction product was measured at 490 nm using a multiwell spectrophotometer.

### Determination of collagen 1A1 synthesis

Collagen synthesis was determined by measuring the concentration of P1NP in the cell medium as described [14, 15]. Briefly, normal human skin fibroblasts were cultured for 48 hours with the indicated compounds. Then, cell-free supernatants were collected and analyzed for P1NP according to the manufacturer’s instructions (P1NP-Elecsys assay, Roche Pharmaceuticals, Grenzach-Wyhlen, Germany). An antigen/antibody complex is formed by addition of biotin-labeled anti-P1NP and Ru(bpy)_3_^2+^-labeled anti-P1NP. Consecutively, the antigen/antibody complex becomes bound to streptavidin-coated magnetic micro-particles added to the sample. The micro-particles were enriched by magnetic separation. After several washes the chemiluminescence is measured with a photomultiplier (Elecsys, Roche).

### Collagen 1A1 promoter transactivation assay

The human collagen 1A1 promoter constructs spanning -2300/+18 and -205/+18, respectively, linked to a luciferase reporter gene in pGL3 basic (Promega, Mannheim, Germany) were a kind gift from Annett Skupin [16]. Constructs were transfected into subconfluently grown human skin fibroblasts by lipofection (Lipofectamin reagent 2000, Invitrogen). In order to standardize transfection efficacy cells were co-transfected with a humanized *Renilla* luciferase vector (phRL, Promega). Cells were treated with 10% milk or 10 ng/ml TGFβ1 for 48 hours. Finally, cells were lysed and luciferase activities of both luciferases were detected separately, using the Dual-Luciferase Reporter Assay System (Promega) and a luminometer (Berthold, Bad Wildbad, Germany).

### Immunoblotting

For detection of activated PKB/Akt, p44/42, p38, STAT6 and total PKB/Akt and β-actin cells were lysed in 100 μl SDS sample buffer (62.5 mM Tris-HCl [pH 6.8], 2% SDS,10% glycerol, 50 mM DTT, 0.1% bromphenol blue), sonicated, boiled for 5 min, and separated on SDS-polyacrylamide gels. Consecutively, proteins were immunoblotted to a PVDF membrane. The membrane was blocked in blocking buffer (TBS [pH 7.6], 0.1% Tween-20, 5% nonfat dry milk) for at least 3 hours at 4°C followed by incubation with the primary antibody in TBS (pH 7.6), 0.05% Tween-20, and 5% BSA. The bound primary antibodies were detected using anti-mouse IgG-horseradish peroxidase conjugate and visualized with the LumiGLO detection system (Cell Signalling).

### Presentation of data and statistical analysis

All data are presented as mean values ± standard deviation. Statistical significance of the data was calculated by the ANOVA-test (BIAS, Frankfurt, Germany). Each set of data was related to the referring untreated controls. To estimate effects of inhibitors or blocking antibodies data were related to positive controls treated with bovine milk and mouse IgG (mIgG). Differences were considered significant at p < 0.05 indicated by an asterisk.

## Results

### Bovine milk induces proliferation

**Fig 1:**
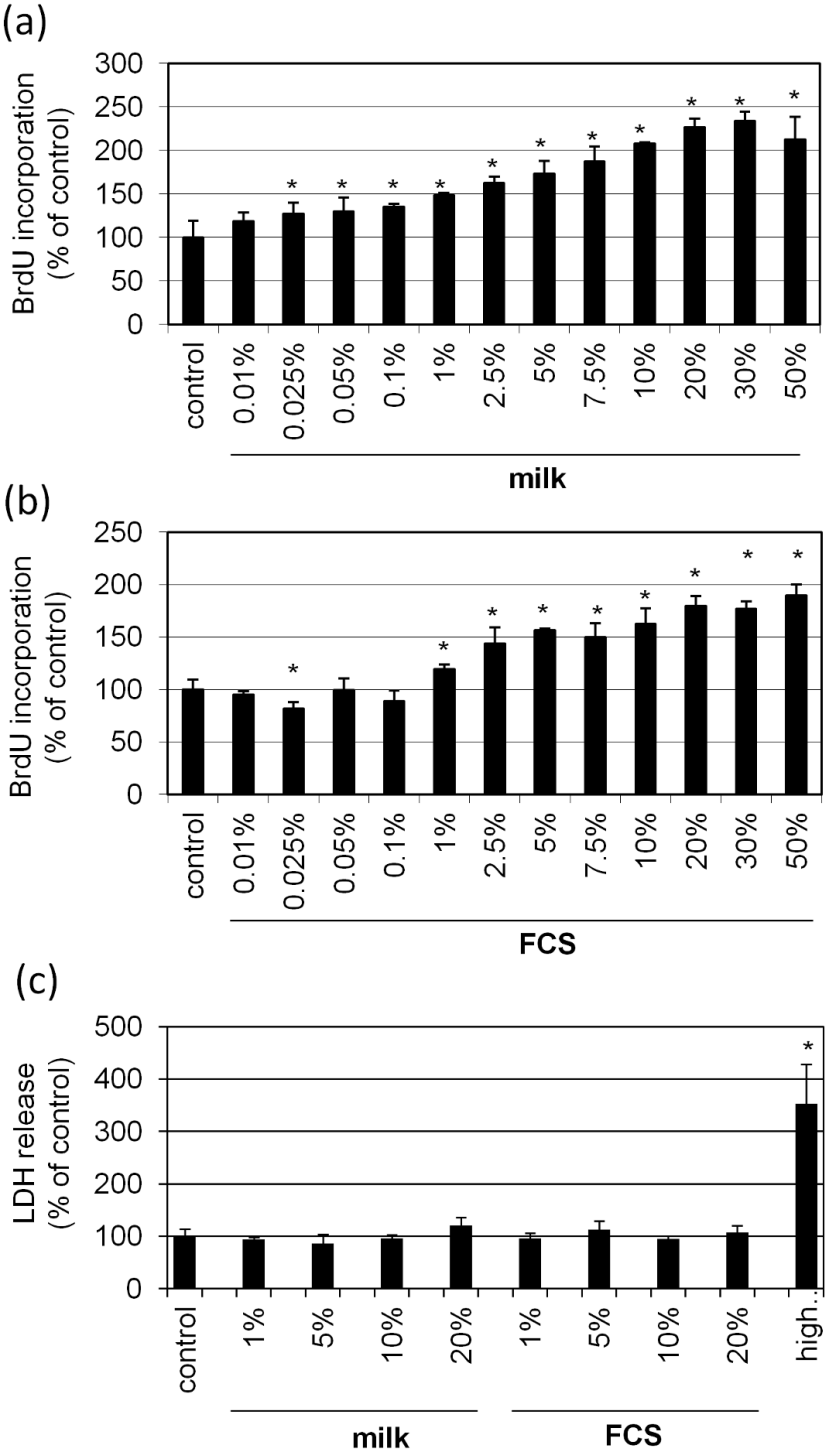
Effect of bovine milk on DNA synthesis and membrane integrity. Normal human skin fibroblasts were cultured in medium supplemented with increasing concentrations of **(a)** bovine milk or **(b)** FCS for control. After 24 hours the incorporation of BrdU in the DNA was determined. Likewise, the liberation of LDH as a marker of membrane integrity was determined after incubation with **(c)** bovine milk or FCS. Complete release of LDH was achieved by treatment with 1% Triton X-100 (high control). Each bar represents the mean of 4 independent experiments. Standard deviations are indicated. Data were compared to untreated controls. *p<0.05.

**Fig 2:**
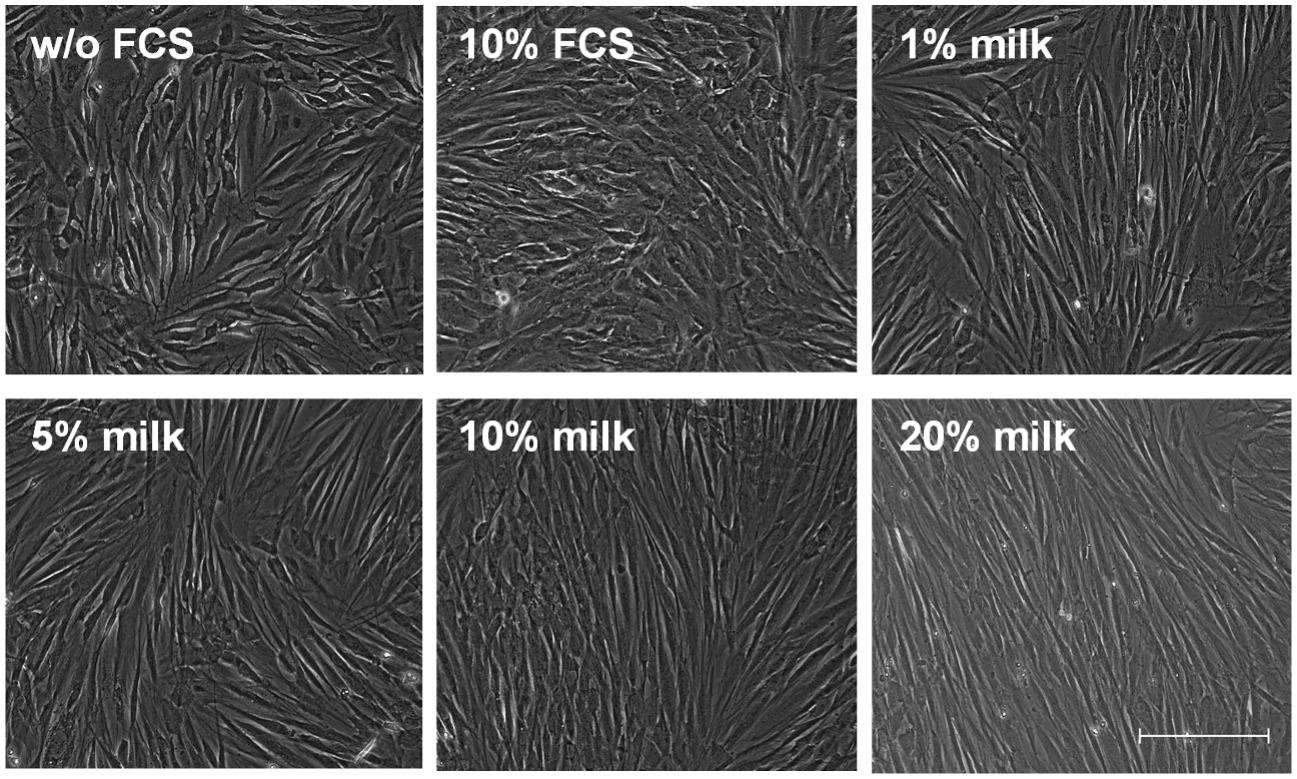
Bovine milk changes cell morphology. Normal human skin fibroblasts were cultured in regular medium without FCS or in the presence of 10% FCS, 1%, 5%, 10% and 20% bovine milk for 24 hours. Representative photographs are shown. Bar = 200μM

Normal human fibroblasts were exposed to bovine milk to test the impact on basal cell parameters such as proliferation and morphology. It was found that substitution of FCS by bovine milk leads to a concentration dependent increase of proliferation compared to cells held in the absence of FCS (Fig 1a). Already at a concentration of 0,025% of bovine milk a significant growth promoting effect was measured. A maximum induction of 2,3-fold was observed in the presence of 30% bovine milk. The calculated EC50 value was reached at 2,5% milk. Additional experiments comparing preputial and abdominal fibroblasts showed similar results (S1 Fig). The milk-induced effect was compared to regular supplementation with FCS (Fig 1b). The figure shows a modest growth-promoting effect at 1% FCS reaching maximum induction of 1,9-fold at 50% FCS with an EC50 at 3% FCS. In order to regard toxic effects LDH levels were measured (Fig 1c). Pretrials demonstrate significant amounts of LDH in medium containing milk or FCS (data not shown). These values become subtracted from supernatants derived from cell cultures. The data shown indicate no cytotoxic effect of either milk or FCS on fibroblasts. Corrobatively, experiments measuring cytoplasmic histone-associated DNA fragments as markers for apoptosis showed no upregulation by milk (data not shown). Moreover, the effect of bovine milk on cell morphology was tested (Fig 2). Replacement of FCS by milk induces a stretched, more spindle-like, cell morphology. In addition, the cells become uniformly orientated like fish in a swarm.

### Bovine milk induces collagen 1A1

**Fig 3:**
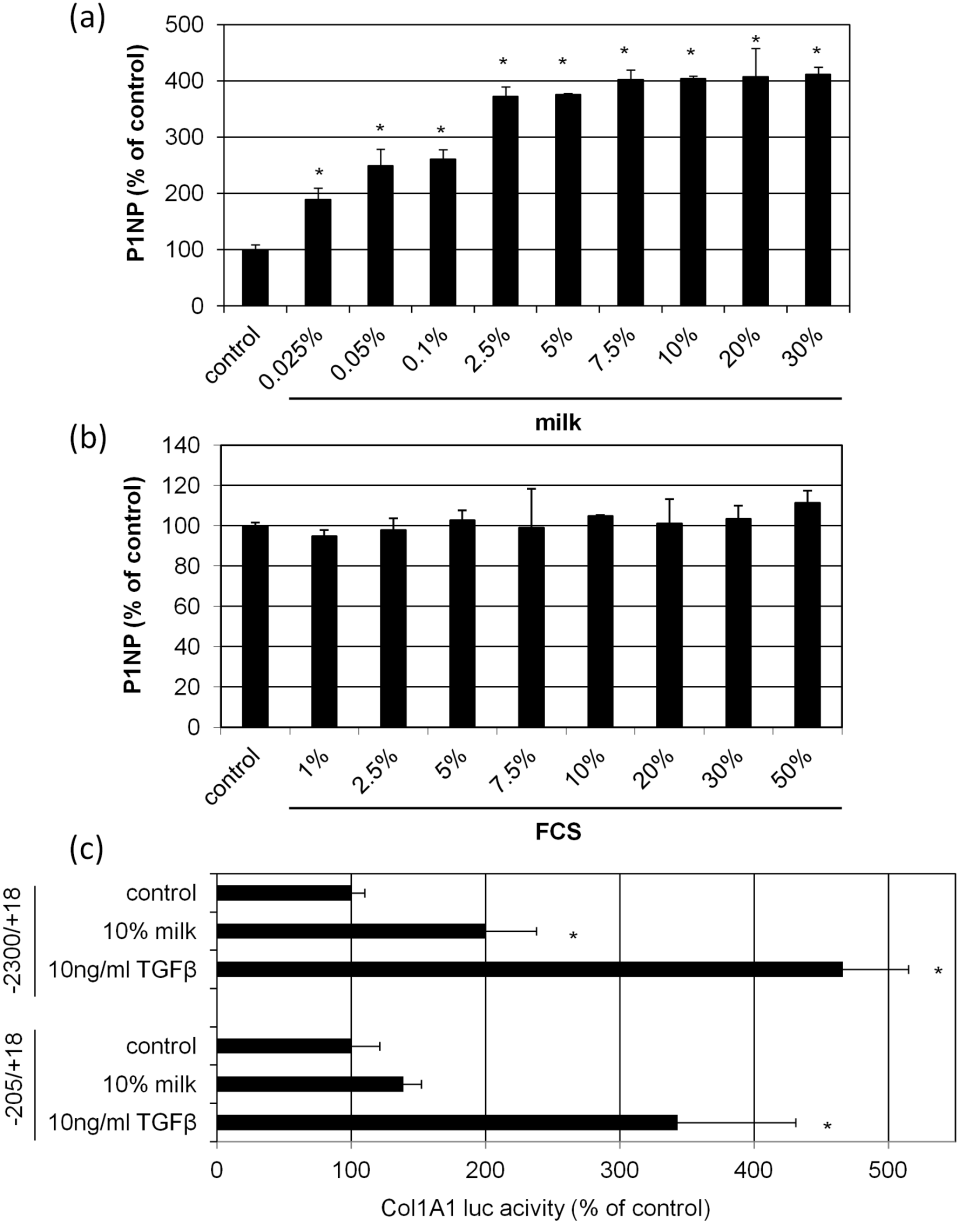
Bovine milk stimulates collagen synthesis and promoter transactivation. Normal human skin fibroblasts were cultured in medium supplemented with increasing concentrations of **(a)** bovine milk or **(b)** FCS for control. After 48 hours the amount of P1NP in the supernatants was measured. **(c)** Analysis of two collagen 1A1 promoter-based luciferase constructs (-2300/+18, -205/+18) in human fibroblasts. After transfection cells were treated with 10% bovine milk or TGFβ1 as positive control for 48 hours. Cells held under FCS-free conditions served as control. Transfection efficiacy was controlled by co-transfection with a humanized Renilla luciferase vector (phRL). Each bar represents the mean of 3 independent experiments. Standard deviations are indicated. Data were compared to untreated controls. *p<0.05

The collagen1A1 synthesis was quantified by determining the amount of pro-collagen type 1 N-terminal pro-peptide (P1NP). Supplementation of the basal medium with bovine milk showed a concentration dependent increase of P1NP to about 4-fold after 48 hours with an EC50 at 0,09% bovine milk (Fig 3a). In all experiments shown in this paper the induction of P1NP by bovine milk was significant, but varied, as expected for biological systems. The body areas where cells were obtained and the donor age may be important parameters for the degree of P1NP induction by bovine milk. Of note, experiments performed with increasing FCS-concentrations showed no significant impact on collagen synthesis (Fig 3b) In order to corroborate the effects of bovine milk on collagen synthesis, promoter transactivation assays were performed (Fig 3c). As positive control served TGFβ1, a well known inductor of collagen synthesis [17, 18]. Our data show significant promoter transactivation for both, TGFβ1 and 10% bovine milk, in a promoter construct spanning from -2300bp to + 18bp. Using a deletion mutant devoid of STAT6 binding motifs (#1–5,6,7) spanning a sequence from -205bp to +18bp [16] TGFβ1 still induces significant promoter transactivation whereas 10% bovine milk failed. These results support findings describing the relevance of STAT6 in collagen promoter transactivation [16].

### Activation of signaling molecules by bovine milk

**Fig 4:**
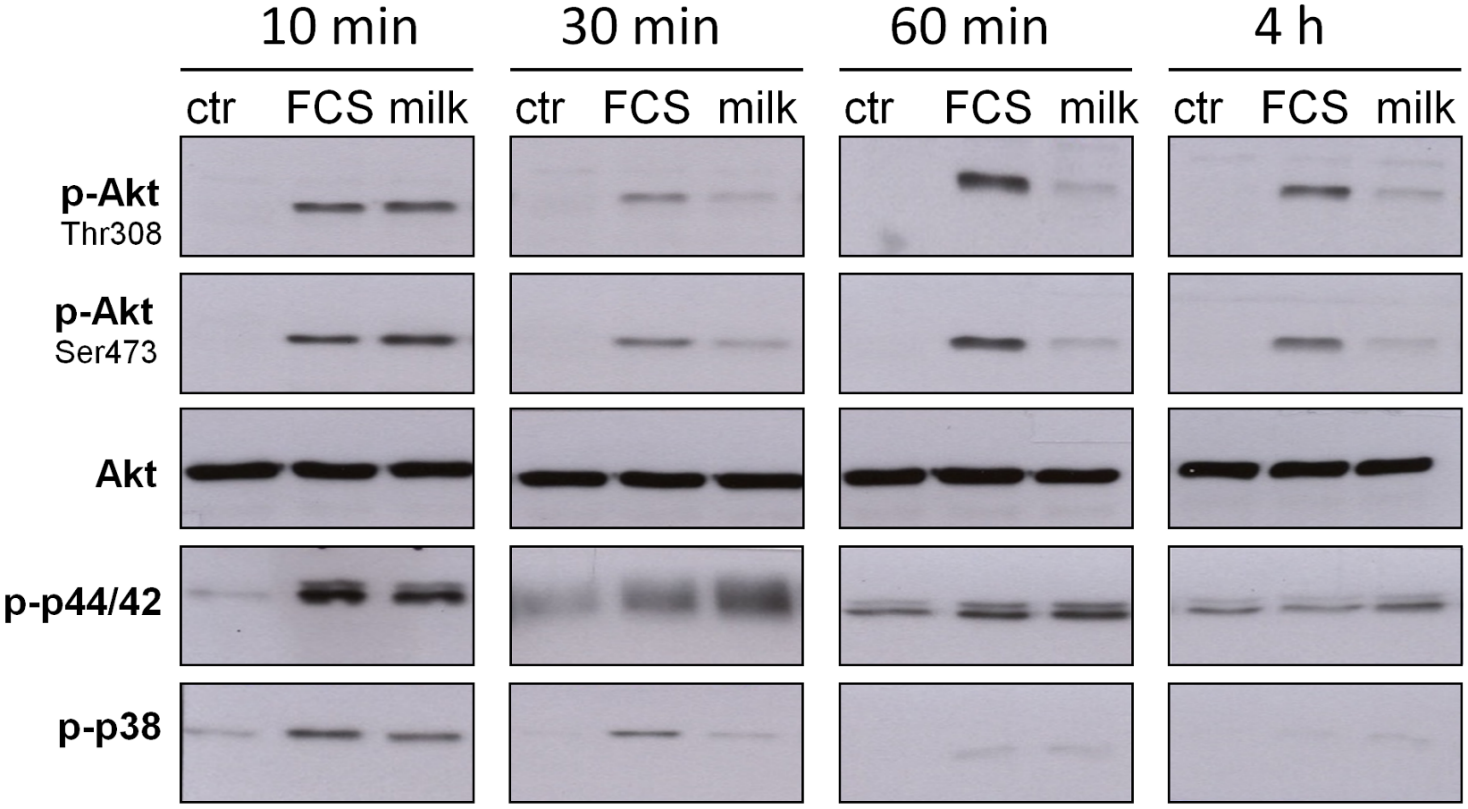
Bovine milk activates signaling molecules. Time course of PKB/Akt, p44/42 and p38 phosphorylation in human fibroblasts. After serum starvation for 24 hours cells were treated with either 10% FCS or 10% bovine milk for 10, 30, 60 minutes or 4 hours. Cells held under FCS-free conditions served as control (ctr). Proteins were obtained as described in “Materials and Methods”, and Western blotting was performed with phosphospecific antibodies. Equal loading was monitored by using antibodies directed against total PKB/Akt. The blot shows representative results. (n = 3).

In order to identify signaling pathways involved in collagen induction by bovine milk fibroblasts were treated with 10% bovine milk for 10, 30, 60 minutes and 4 hours, then, harvested and subjected to Western blot analysis (Fig 4). Using phosphospecific antibodies against key factors in cell signaling revealed a fast and strong activation of PKB/Akt, p44/42 and p38 within 10 minutes. This activation was similar for FCS. Looking at PKB/Akt the milk-induced activation decreased faster with time than in the case of FCS. Of note, 4 hours after adding FCS a significant activation is still present. The phosphorylation of p44/42 by bovine milk and FCS was similar, lasting for at least 60 minutes. Phosphorylation of p38 after 30 minutes was more distinct for FCS than for bovine milk. Longer incubations showed only marginal signals for both compounds.

### Soluble factors in bovine milk induce collagen synthesis

**Fig 5:**
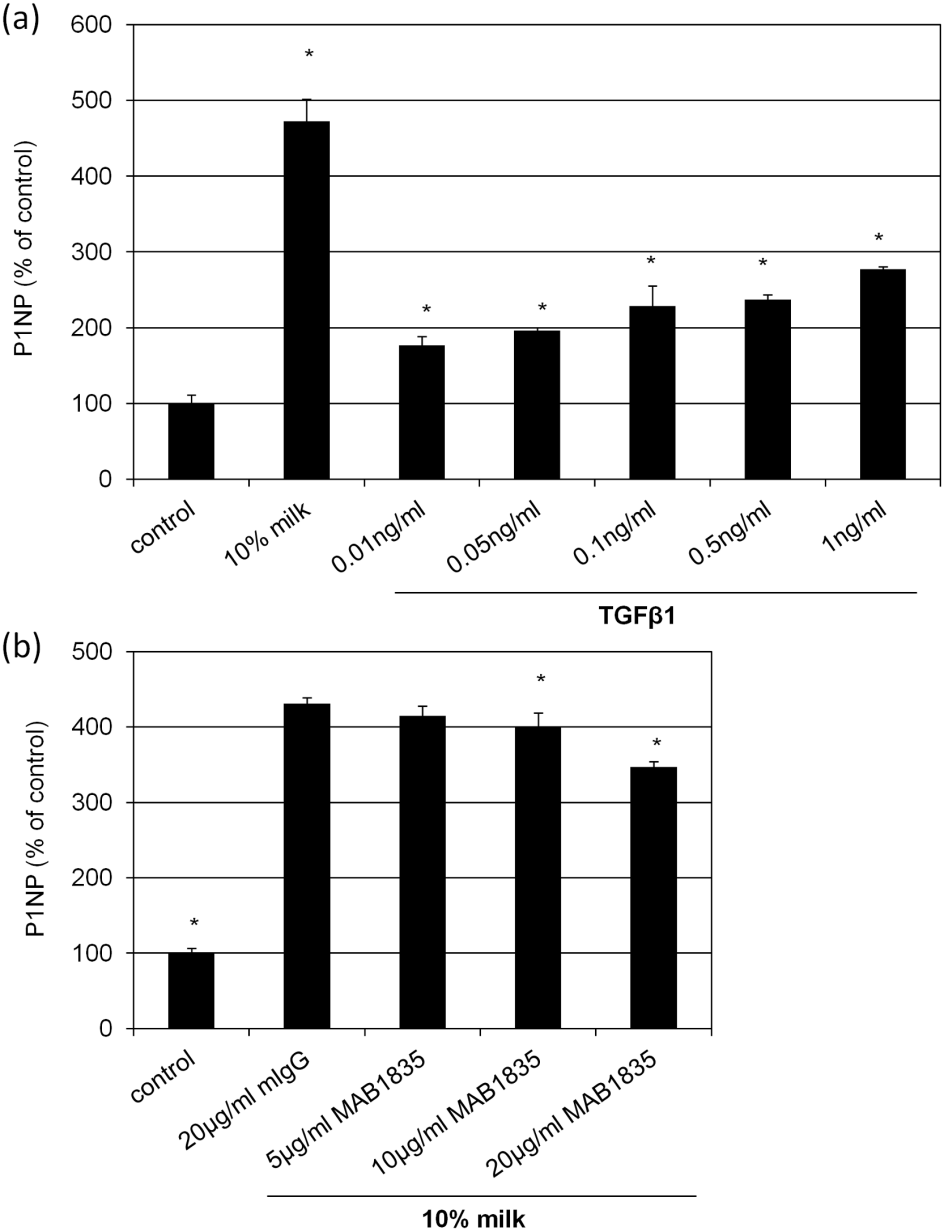
TGFβ1 regulates collagen synthesis. Human skin fibroblasts were treated **(a)** with 10% bovine milk or with 0.01, 0.05, 0.1, 0.5, 1.0 ng/ml TGFβ1 or **(b)** with 10% milk containing 5, 10, 20 μg/ml TGFβ1,2,3 neutralizing antibody MAB1835. Cells held under FCS-free conditions served as control. After 48 h the amount of P1NP in the supernatants was measured. Each bar represents the mean of 3 independent experiments. Standard deviations are indicated. Data of (a) were compared to cells held under FCS-free conditions, and (b) treated with 10% milk and 20 μg/ml mIgG. *p<0.05

**Fig 6:**
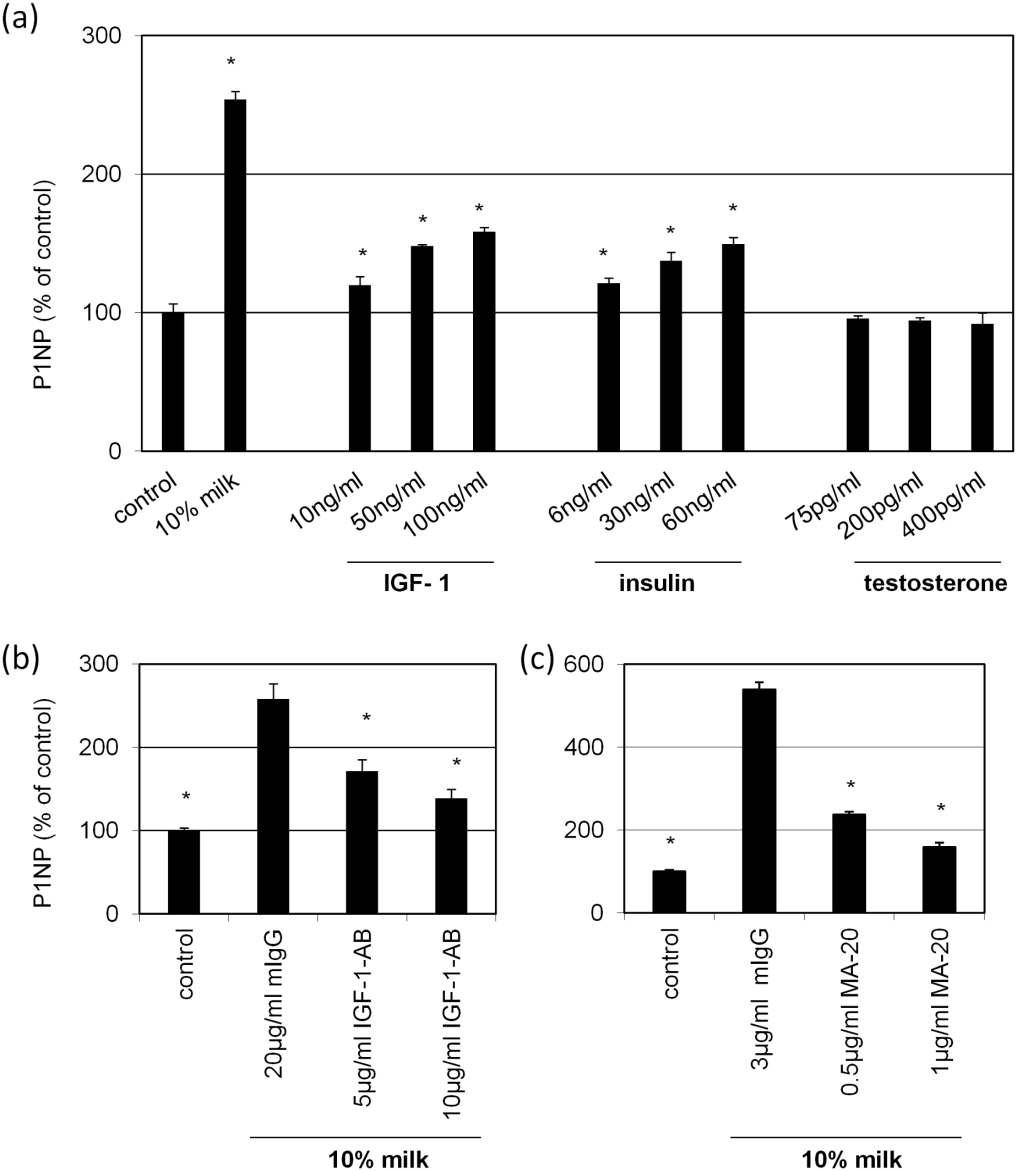
Impact of IGF-1, insulin and testosterone on collagen synthesis. Human skin fibroblasts were treated **(a)** with 10% bovine milk or with 10, 50, 100 ng/ml IGF-1, 6, 30, 60ng/ml insulin or with 75, 200, 400pg/ml testosterone or **(b)** with 10% bovine milk containing 5 and 10 μg/ml IGF-1 neutralizing antibody #5119–100 or **(c)** with 10% bovine milk containing 0.5 and 1 μg/ml MA-20, a functional blocking antibody against the insulin receptor. Cells held under FCS-free conditions served as control. After 48 hours the amount of P1NP in the supernatants was measured. Each bar represents the mean of 3 independent experiments. Standard deviations are indicated. Data of (a) were compared with cells held under FCS-free conditions, and (b,c) treated with 10% milk and mIgG. *p<0.05

One of the most likely candidate factors within milk responsible for milk-induced collagen synthesis is TGFβ1 which was shown to increase collagen synthesis [17, 18]. The effect of recombinant TGFβ1 on P1NP is given in Fig 5a featuring a concentration dependent increase. In order to eliminate TGFβ1 from milk the neutralizing antibody MAB1835, specific for TGF1, 2 and 3 subsets, was used (Fig 5b). Of note, only at high concentrations (>10 μg/ml) a slight but significant suppression of milk-induced P1NP synthesis was observed. However, at 20 μg/ml MAB1835 the suppression was only 20% compared to the control indicating that TGFβ1 is not the main component in milk responsible for collagen synthesis. In order to test other candidate molecules present in milk cells were stimulated with IGF-1, insulin and testosterone (Fig 6a). For IGF-1 and insulin a concentration dependent increase of P1NP synthesis was found. However, both compounds failed to reach the level measured with bovine milk. Moreover, testosterone showed no stimulatory effect on collagen synthesis. To further substantiate the role of IGF-1 and insulin, milk was supplemented with neutralizing antibody against IGF-1 (Fig 6b) and the insulin receptor (Fig 6c). The milk-inducing effect on collagen synthesis was significantly reduced in the presence of anti-IGF-1 and MA-20, respectively. This indicates that both, IGF-1 and particularly insulin, play a major role in collagen synthesis induced my bovine milk.

### Activation of STAT6 by bovine milk mediates collagen synthesis

**Fig 7:**
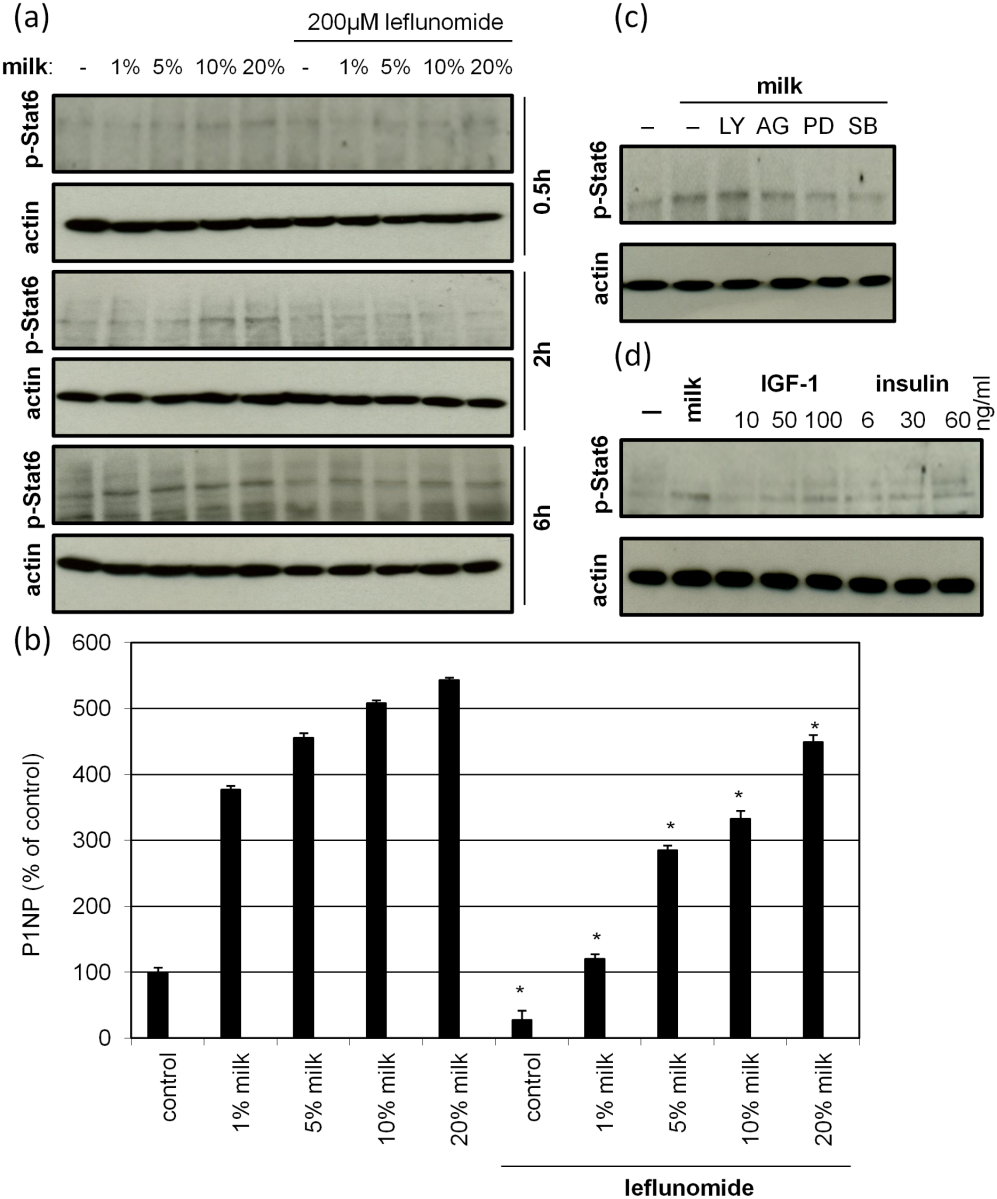
Bovine milk regulates collagen synthesis via STAT6. **(a)** Human skin fibroblasts were treated with 1, 5, 10 and 20% bovine milk with or without 200 μM leflunomide, an inhibitor of p-STAT6. After 0,5, 2 and 6h cell lysates were examined for p-STAT6 induction by Western blot. **(b)** Cells were treated with 1, 5, 10 and 20% bovine milk with or without 200μM leflunomide. After 24 hours the amount of P1NP in the supernatants was measured. Each bar represents the mean of 3 independent experiments. Standard deviations are indicated. Data were compared to cells held under FCS-free conditions. *p<0.05. **(c)** Cells were treated with 20% bovine milk in the presence of specific kinase inhibitors (20 μM LY294002, 500 nM AG1478, 100 μM PD98059, 20 μM SB203580). After 6h cell lysates were examined for p-STAT6 induction. **(d)** Cells were treated with either 20% bovine milk or with increasing concentrations of IGF-1 or insulin. After 6h cell lysates were examined for p-STAT6 induction. Equal loading was monitored by using antibodies directed against actin. The blots show representative results. (n = 3).

Data derived from promoter transactivation assays suggest a role of STAT6 in the collagen promoting effect of bovine milk (Fig 3c). This observation was substantiated by showing that the addition of bovine milk led to a concentration and time dependent induction of p-STAT6 phosphorylation (Fig 7a). Using leflunomide, an inhibitor of p-STAT6 [13], reverted the milk-induced p-STAT6 phosphorylation corroborating the aforementioned effect of this agent (Fig 7a). In a pretrial we tested that leflunomide has no cytotoxic impact at concentrations used (S2 Fig). Corroborating the effect of p-STAT6 in our model we show a significant inhibition of milk-induced collagen synthesis in the presence of leflunomide (Fig 7b). In order to learn more about upstream signaling we performed experiments using specific kinase inhibitors. These experiments feature significant fainter bands of p-STAT6 in cells treated with PD98059, and particularly with SB203580 (Fig 7c). These data suggest a contribution of the MEK1 and p38 pathway. Finally, it was investigated if IGF-1 and insulin, identified as collagen inductors in bovine milk, were capable to induce STAT6 phosphorylation. As shown in Fig 7d IGF-1 induces phosphorylation in a concentration dependent manner, insulin shows no such effect.

## Discussion

Milk is a cocktail of bioactive compounds. Besides lactose, the distinctive milk disaccharide, it contains considerable amounts of amino acids, minerals and vitamins [19], a plethora of about 400 different fatty acids [20] and also large amounts of microRNAs [21, 22]. Moreover, endocrine factors such as steroid and peptide hormones are present. Additionally, bovine milk contains various growth factors with TGFβ-α/β and IGF-1 as key factors [23]. All these factors are considered to regulate the neonatal body function. Interestingly, there is evidence that milk consumption increases also IGF-1 serum levels [24, 25] by possibly a direct transfer from the gastrointestinal tract into systemic circulation [26]. Therefore, it could be speculated that some milk derived factors may take effect also systemically. By topical application, however, these factors can directly act on skin cells, particularly under conditions with impaired skin barrier.

Our results demonstrate that bovine milk modulates basic physiologic factors of skin fibroblasts. In particular, we observed an induction of proliferation accompanied with morphological changes. Specifically, cells become unidirectionally stretched, which fits well to the growth-índucing effect, as cell stretch was identified as potent inductor of proliferation and proliferation-associated kinases [27, 28]. However, the key finding of this paper describes the strong induction of collagen 1A1 synthesis in response to bovine milk. In order to identify factors within milk responsible for this effect, TGFβ1 seemed to be the most likely candidate molecule, known to induce collagen synthesis [29]. Of note, medium supplemented with TGFβ1 showed only meager induction of P1NP compared with bovine milk. Likewise, neutralization of TGFβ1 within milk resulted only in a modest reduction of milk-induced collagen synthesis. This finding indicates that TGFβ1 plays at least only a minor part in collagen induction. Next, IGF-1, another collagen-inducing growth factor [30], was tested. In contrast to the aforementioned experiments with TGFβ1, neutralization of IGF-1 showed a strong inhibition of the collagen promoting effect of bovine milk underlining the significance of this bioactive factor in this context. The stimulatory effect of IGF-1 can be modulated by carrier proteins belonging to the IGF binding protein (IGFBP) family which are present in bovine milk [23] and also known to be produced by fibroblasts themselves [31]. Moreover, we investigated the role of insulin on collagen synthesis. Similar to IGF-1, insulin given to fibroblasts increased collagen synthesis in fibroblasts which is in concert with previous findings [32–34]. Neutralization of insulin within milk by an antibody directed against the insulin receptor (MA-20) caused the strongest suppression of collagen synthesis followed by IGF-1 and TGFβ1, having only a weak effect. However, insulin given to the cells does not activate STAT6 indicating that also other signaling molecules are involved in the regulation of collagen synthesis. Promotor analysis of the Col1A1 gene showed also binding motifs for other transcription factors such as AP2, NF1 and SP1 [1] which may contribute to the observed insulin effect. Besides growth factors bovine milk contains considerable amounts of sex steroid hormones including progesterone and its metabolite testosterone [35]. Others have shown that some anabolic steroid hormones stimulated collagen synthesis [36]. Therefore, the effect of testosterone, present in a concentration ranging between 50 and 150pg/ml in bovine milk [37], was tested. Our data show no regulation of collagen synthesis by testosterone.

Regarding signal transduction we found STAT6 being an important factor. In particular we provide evidence that collagen promoter transactivation in response to milk is sensitive to the presence of STAT6 binding sites. A similar finding was reported for IL4 signalling [16]. Moreover, this finding was substantiated by showing that milk phosphorylates STAT6. *Vice versa*, Leflunomide, a STAT6 inhibitor, diminished milk-induced collagen synthesis. In parallel a transient activation of PKB/Akt, p44/42 and p38 by bovine milk was found. Specific inhibitors targeting the p44/42 and p38 pathway inhibited milk-induced STAT6 phosphorylation, whereas PKB/Akt does not seem to be involved. Although, STAT6 is prototypically phosphorylated in a IL-4 and IL-13 dependent manner [38] our result show also activation upon IGF-1 stimulation which is concert with recent findings [39]. Assuming that STAT6 is a key player in adaptive immunity [40, 41], it could be speculated that exposure of skin to milk-derived factors such as IGF-1 may have also an effect in this context.

In sum, our data show that bovine milk which is traditionally used for skin care offers significant effects on skin fibroblasts. Particularly, the strong induction of proliferation and collagen synthesis makes this body fluid an interesting subject for clinical tests. It could be speculated from our findings that clinical conditions featuring a downregulated fibroblast metabolism, which prototypically occurs in UV-aged skin and after long-term corticosteroid treatment, may benefit from topical agents containing milk-derived factors.

## Supporting Information

S1 Fig Bovine milk induces DNA synthesis in preputial and abdominal fibroblast to the same extend. Preputial and abdominal normal human skin fibroblasts were cultured in medium supplemented with increasing concentrations of bovine milk or 10% FCS as positive control. After 24 hours the incorporation of BrdU in the DNA was determined. Each bar represents the mean of 4 independent experiments. Standard deviations are indicated. Data were compared to untreated controls. *p<0.05.
(TIF)

S2 Fig Leflunomide offer no distinct cytotoxicity on human skin fibroblasts. Cell were treated with 100, 200 and 300 μM leflunomide. After 24h LDH content was determined in cell free supernatants. Complete release of LDH was achieved by treatment with 1% Triton X-100 (high). Each bar represents the mean of 4 independent experiments. Standard deviations are indicated. Data were compared to untreated controls. *p<0.05.
(TIF)

## References

[1] Hanson LA, Ahlstedt S, Andersson B, Carlsson B, Fallstrom SP, Mellander L, et al Protective factors in milk and the development of the immune system. Pediatrics. 1985;75(1 Pt 2):172–6. 3880886

[2] Melnik B. Milk consumption: aggravating factor of acne and promoter of chronic diseases of Western societies. JDtschDermatolGes. 2009;7(4):364–70. doi: DDG07019 [pii]; 10.1111/j.1610-0387.2009.07019.x 19243483

[3] Adebamowo CA, Spiegelman D, Berkey CS, Danby FW, Rockett HH, Colditz GA, et al Milk consumption and acne in teenaged boys. Journal of the American Academy of Dermatology. 2008;58(5):787–93. Epub 2008/01/16. 10.1016/j.jaad.2007.08.049 . 18194824 PMC4391699

[4] Adebamowo CA, Spiegelman D, Danby FW, Frazier AL, Willett WC, Holmes MD. High school dietary dairy intake and teenage acne. Journal of the American Academy of Dermatology. 2005;52(2):207–14. Epub 2005/02/05. 10.1016/j.jaad.2004.08.007 . 15692464

[5] Michaëlsson K, Wolk A, Langenskiöld S, Basu S, Warensjö Lemming E, Melhus H, et al Milk intake and risk of mortality and fractures in women and men: cohort studies2014 2014-10-28 23:31:15. 10.1136/bmj.g6015 PMC4212225 25352269

[6] Ganmaa D, Wang PY, Qin LQ, Hoshi K, Sato A. Is milk responsible for male reproductive disorders? MedHypotheses. 2001;57(4):510–4. 10.1054/mehy.2001.1380;S0306-9877(01)91380-5 [pii]. 11601881

[7] Klein J, Fasshauer M, Ito M, Lowell BB, Benito M, Kahn CR. beta(3)-adrenergic stimulation differentially inhibits insulin signaling and decreases insulin-induced glucose uptake in brown adipocytes. The Journal of biological chemistry. 1999;274(49):34795–802. Epub 1999/11/27. . 10574950 10.1074/jbc.274.49.34795

[8] Belury MA. Inhibition of carcinogenesis by conjugated linoleic acid: potential mechanisms of action. J Nutr. 2002;132(10):2995–8. Epub 2002/10/09. . 12368384 10.1093/jn/131.10.2995

[9] Zhou XR, Sun CH, Liu JR, Zhao D. Dietary conjugated linoleic acid increases PPAR gamma gene expression in adipose tissue of obese rat, and improves insulin resistance. Growth Horm IGF Res. 2008;18(5):361–8. Epub 2008/02/29. 10.1016/j.ghir.2008.01.001 . 18304850

[10] Kumura H, Sawada T, Oda Y, Konno M, Kobayashi K. Potential of polar lipids from bovine milk to regulate the rodent dorsal hair cycle. J Dairy Sci. 2012;95(7):3629–33. Epub 2012/06/23. 10.3168/jds.2011-5304 . 22720920

[11] Dennis CL, Jackson K, Watson J. Interventions for treating painful nipples among breastfeeding women. The Cochrane database of systematic reviews. 2014;12:Cd007366 Epub 2014/12/17. 10.1002/14651858.CD007366.pub2 . 25506813 PMC10885851

[12] Gustafsson L, Leijonhufvud I, Aronsson A, Mossberg AK, Svanborg C. Treatment of skin papillomas with topical alpha-lactalbumin-oleic acid. NEnglJMed. 2004;350(26):2663–72. 10.1056/NEJMoa032454;350/26/2663 [pii]. 15215482

[13] Olsan EE, Mukherjee S, Wulkersdorfer B, Shillingford JM, Giovannone AJ, Todorov G, et al Signal transducer and activator of transcription-6 (STAT6) inhibition suppresses renal cyst growth in polycystic kidney disease. Proc Natl Acad Sci U S A. 2011;108(44):18067–72. Epub 2011/10/26. 10.1073/pnas.1111966108 ; PubMed Central PMCID: PMCPmc3207695. 22025716 PMC3207695

[14] Harrison CA, Gossiel F, Bullock AJ, Sun T, Blumsohn A, Mac NS. Investigation of keratinocyte regulation of collagen I synthesis by dermal fibroblasts in a simple in vitro model. BrJDermatol. 2006;154(3):401–10. doi: BJD7022 [pii]; 10.1111/j.1365-2133.2005.07022.x 16445767

[15] Zoller NN, Kippenberger S, Thaci D, Mewes K, Spiegel M, Sattler A, et al Evaluation of beneficial and adverse effects of glucocorticoids on a newly developed full-thickness skin model. ToxicolIn Vitro. 2008;22(3):747–59. doi: S0887-2333(07)00334-7 [pii]; 10.1016/j.tiv.2007.11.022 18249522

[16] Buttner C, Skupin A, Rieber EP. Transcriptional activation of the type I collagen genes COL1A1 and COL1A2 in fibroblasts by interleukin-4: analysis of the functional collagen promoter sequences. JCell Physiol. 2004;198(2):248–58. 10.1002/jcp.10395 14603527

[17] Raghow R, Postlethwaite AE, Keski-Oja J, Moses HL, Kang AH. Transforming growth factor-beta increases steady state levels of type I procollagen and fibronectin messenger RNAs posttranscriptionally in cultured human dermal fibroblasts. JClinInvest. 1987;79(4):1285–8. 10.1172/JCI112950 PMC424335 3470308

[18] Varga J, Rosenbloom J, Jimenez SA. Transforming growth factor beta (TGF beta) causes a persistent increase in steady-state amounts of type I and type III collagen and fibronectin mRNAs in normal human dermal fibroblasts. BiochemJ. 1987;247(3):597–604. 3501287 10.1042/bj2470597 PMC1148454

[19] Jenness R. Proceedings: Biosynthesis and composition of milk. The Journal of investigative dermatology. 1974;63(1):109–18. Epub 1974/07/01. . 4600634 10.1111/1523-1747.ep12678111

[20] Mansson HL. Fatty acids in bovine milk fat. Food NutrRes. 2008;52 10.3402/fnr.v52i0.1821;1821 [pii]. PMC2596709 19109654

[21] Chen X, Gao C, Li H, Huang L, Sun Q, Dong Y, et al Identification and characterization of microRNAs in raw milk during different periods of lactation, commercial fluid, and powdered milk products. Cell Res. 2010;20(10):1128–37. doi: cr201080 [pii]; 10.1038/cr.2010.80 20548333

[22] Kosaka N, Izumi H, Sekine K, Ochiya T. microRNA as a new immune-regulatory agent in breast milk. Silence. 2010;1(1):7 1758-907X-1-7 [pii]; 10.1186/1758-907X-1-7 20226005 PMC2847997

[23] Blum JW, Baumrucker CR. Insulin-like growth factors (IGFs), IGF binding proteins, and other endocrine factors in milk: role in the newborn. AdvExpMedBiol. 2008;606:397–422. 10.1007/978-0-387-74087-4_16 18183939

[24] Maruyama K, Iso H, Ito Y, Watanabe Y, Inaba Y, Tajima K, et al Associations of food and nutrient intakes with serum IGF-I, IGF-II, IGFBP-3, TGF-b1, total SOD activity and sFas levels among middle-aged Japanese: the Japan Collaborative Cohort study. Asian PacJCancer Prev. 2009;10 Suppl:7–22. 20553076

[25] Hoppe C, Kristensen M, Boiesen M, Kudsk J, Fleischer MK, Molgaard C. Short-term effects of replacing milk with cola beverages on insulin-like growth factor-I and insulin-glucose metabolism: a 10 d interventional study in young men. BrJNutr. 2009;102(7):1047–51. doi: S0007114509338829 [pii]; 10.1017/S0007114509338829 19772696

[26] Sparks AL, Kirkpatrick JG, Chamberlain CS, Waldner D, Spicer LJ. Insulin-like growth factor-I and its binding proteins in colostrum compared to measures in serum of Holstein neonates. JDairy Sci. 2003;86(6):2022–9. doi: S0022-0302(03)73791-6 [pii]; 10.3168/jds.S0022-0302(03)73791-6 12836938

[27] Kippenberger S, Bernd A, Loitsch S, Guschel M, Muller J, Bereiter-Hahn J, et al Signaling of mechanical stretch in human keratinocytes via MAP kinases. JInvest Dermatol. 2000;114(3):408–12. doi: jid915 [pii]; 10.1046/j.1523-1747.2000.00915.x 10692097

[28] Kippenberger S, Loitsch S, Guschel M, Muller J, Knies Y, Kaufmann R, et al Mechanical stretch stimulates protein kinase B/Akt phosphorylation in epidermal cells via angiotensin II type 1 receptor and epidermal growth factor receptor. JBiolChem. 2005;280(4):3060–7. doi: M409590200 [pii]; 10.1074/jbc.M409590200 15545271

[29] Verrecchia F, Chu ML, Mauviel A. Identification of novel TGF-beta /Smad gene targets in dermal fibroblasts using a combined cDNA microarray/promoter transactivation approach. JBiolChem. 2001;276(20):17058–62. 10.1074/jbc.M100754200;M100754200 [pii]. 11279127

[30] Gillery P, Leperre A, Maquart FX, Borel JP. Insulin-like growth factor-I (IGF-I) stimulates protein synthesis and collagen gene expression in monolayer and lattice cultures of fibroblasts. JCell Physiol. 1992;152(2):389–96. 10.1002/jcp.1041520221 1639869

[31] Yateman ME, Claffey DC, Cwyfan Hughes SC, Frost VJ, Wass JA, Holly JM. Cytokines modulate the sensitivity of human fibroblasts to stimulation with insulin-like growth factor-I (IGF-I) by altering endogenous IGF-binding protein production. JEndocrinol. 1993;137(1):151–9. 7684061 10.1677/joe.0.1370151

[32] Kream BE, Smith MD, Canalis E, Raisz LG. Characterization of the effect of insulin on collagen synthesis in fetal rat bone. Endocrinology. 1985;116(1):296–302. 3880543 10.1210/endo-116-1-296

[33] Krupsky M, Fine A, Kuang PP, Berk JL, Goldstein RH. Regulation of type I collagen production by insulin and transforming growth factor-beta in human lung fibroblasts. ConnectTissue Res. 1996;34(1):53–62. 10.3109/03008209609028893 8835848

[34] Rosen DM, Luben RA. Multiple hormonal mechanisms for the control of collagen synthesis in an osteoblast-like cell line, MMB-1. Endocrinology. 1983;112(3):992–9. 6337052 10.1210/endo-112-3-992

[35] Jouan PN, Pouliot Y, Gauthier SF, Laforest JP. Hormones in bovine milk and milk products: A survey. International Dairy Journal. 2006;16(11):1408–14.

[36] Falanga V, Greenberg AS, Zhou L, Ochoa SM, Roberts AB, Falabella A, et al Stimulation of collagen synthesis by the anabolic steroid stanozolol. JInvest Dermatol. 1998;111(6):1193–7. 10.1046/j.1523-1747.1998.00431.x 9856839

[37] Hoffmann B, Rattenberger E. Testosterone Concentrations in Tissue from Veal Calves, Bulls and Heifers and in Milk-Samples. Journal of Animal Science. 1977;45(3):635–41. 903314 10.2527/jas1977.453635x

[38] Takeda K, Kishimoto T, Akira S. STAT6: its role in interleukin 4-mediated biological functions. J Mol Med (Berl). 1997;75(5):317–26. Epub 1997/05/01. . 9181473 10.1007/s001090050117

[39] Osorio EY, Travi BL, da Cruz AM, Saldarriaga OA, Medina AA, Melby PC. Growth factor and Th2 cytokine signaling pathways converge at STAT6 to promote arginase expression in progressive experimental visceral leishmaniasis. PLoS Pathog. 2014;10(6):e1004165 Epub 2014/06/27. 10.1371/journal.ppat.1004165 ; PubMed Central PMCID: PMCPmc4072777. 24967908 PMC4072777

[40] Chen H, Sun H, You F, Sun W, Zhou X, Chen L, et al Activation of STAT6 by STING is critical for antiviral innate immunity. Cell. 2011;147(2):436–46. Epub 2011/10/18. 10.1016/j.cell.2011.09.022 . 22000020

[41] Zeng WP. 'All things considered': transcriptional regulation of T helper type 2 cell differentiation from precursor to effector activation. Immunology. 2013;140(1):31–8. Epub 2013/05/15. 10.1111/imm.12121 ; PubMed Central PMCID: PMCPmc3809703. 23668241 PMC3809703

