# Supplementary figures and images for: STAT6-Dependent Collagen Synthesis in Human Fibroblasts Is Induced by Bovine Milk
Source: PLoS One. 2015 Jul 2;10(7):e0131783. doi: 10.1371/journal.pone.0131783 (PMC4489876; doi:10.1371/journal.pone.0131783)

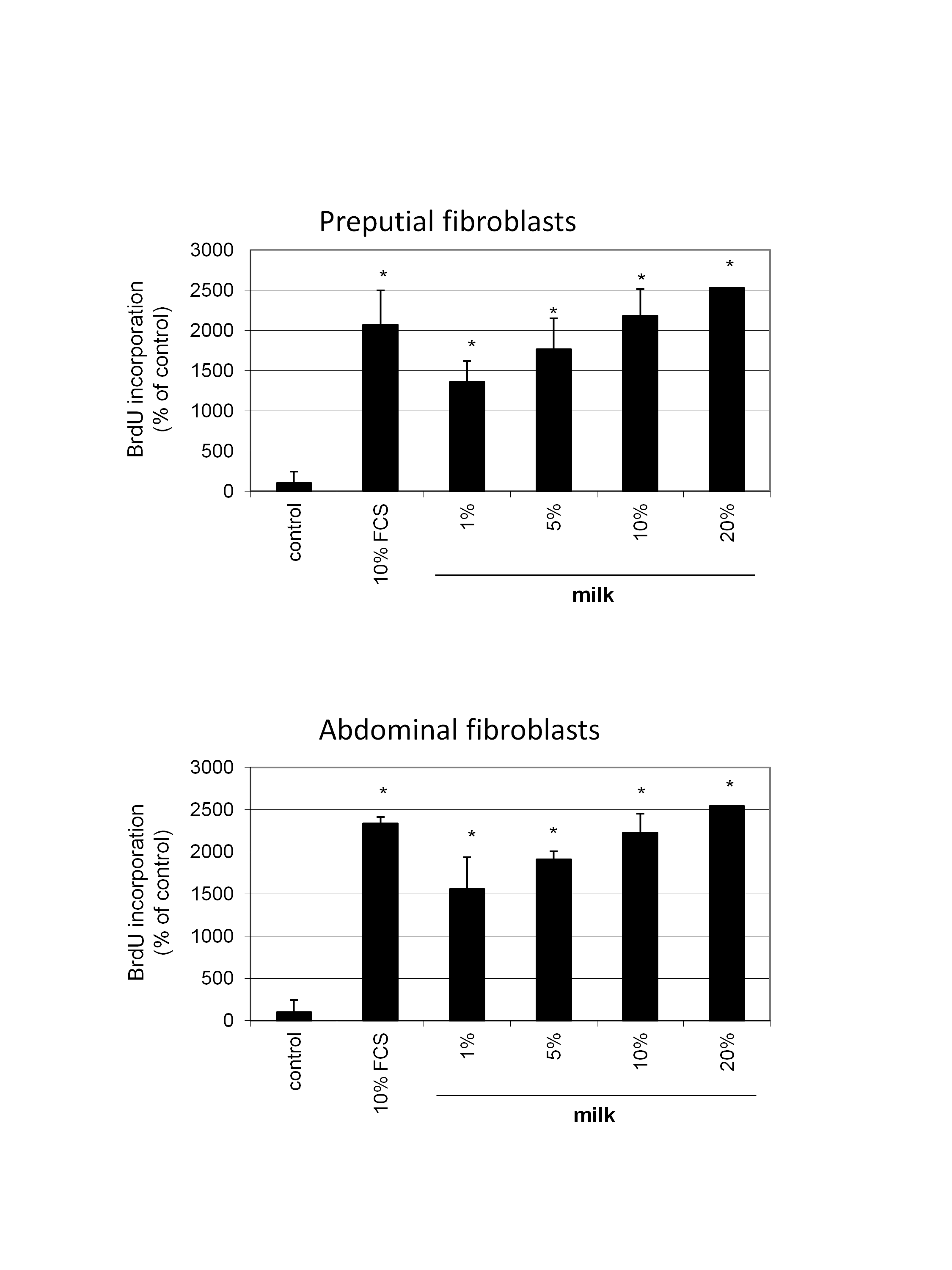

Supplement: S1 Fig — Preputial and abdominal normal human skin fibroblasts were cultured in medium supplemented with increasing concentrations of bovine milk or 10% FCS as positive control. After 24 hours the incorporation of BrdU in the DNA was determined. Each bar represents the mean of 4 independent experiments. Standard deviations are indicated. Data were compared to untreated controls. *p<0.05. (TIF) [file pone.0131783.s001.TIF]

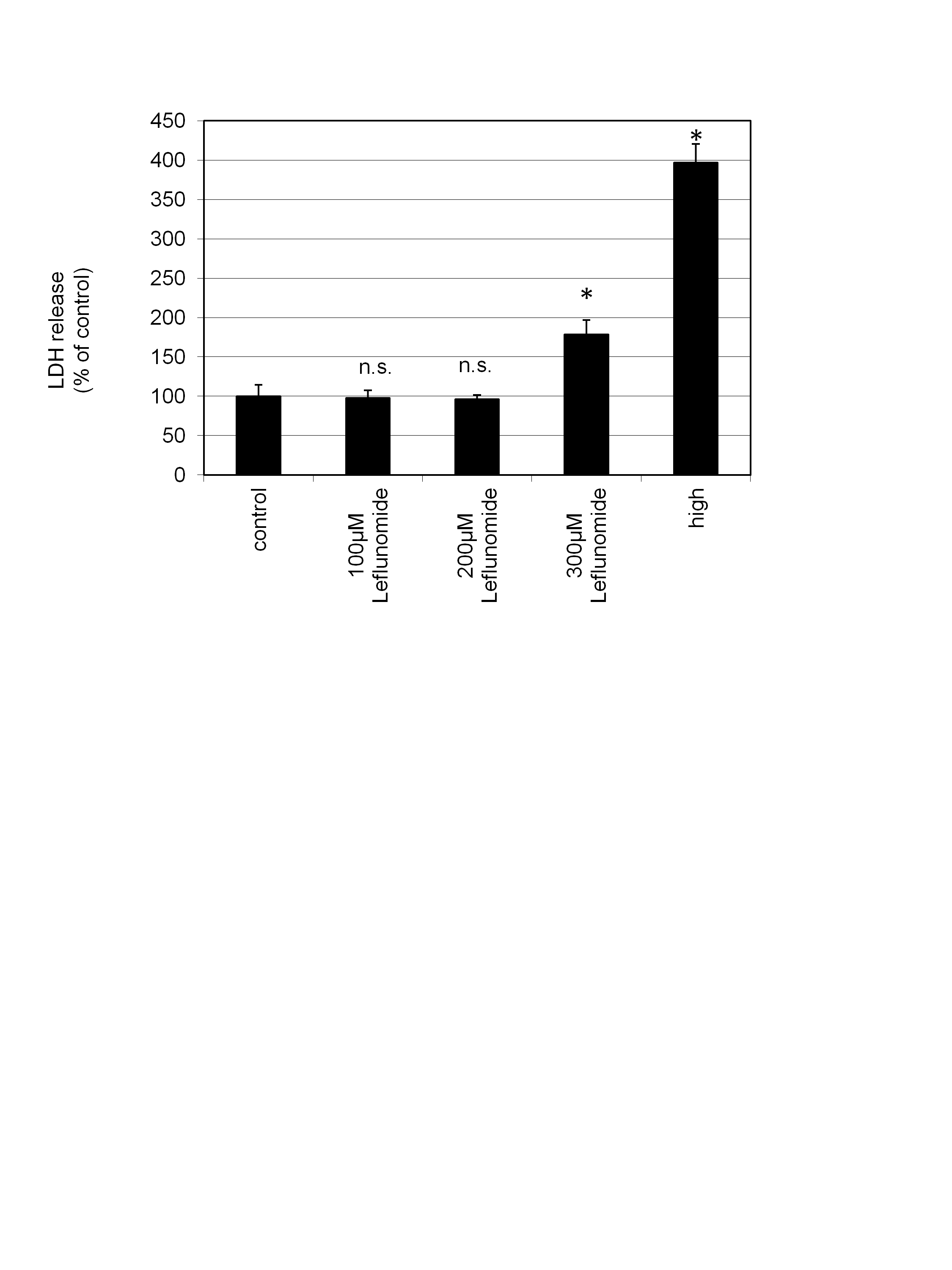

Supplement: S2 Fig — Cell were treated with 100, 200 and 300 μM leflunomide. After 24h LDH content was determined in cell free supernatants. Complete release of LDH was achieved by treatment with 1% Triton X-100 (high). Each bar represents the mean of 4 independent experiments. Standard deviations are indicated. Data were compared to untreated controls. *p<0.05. (TIF) [file pone.0131783.s002.TIF]
